# Hesitant avoidance while walking: an error of social behavior generated by mutual interaction

**DOI:** 10.3389/fpsyg.2015.01013

**Published:** 2015-07-21

**Authors:** Motoyasu Honma, Shinichi Koyama, Mitsuru Kawamura

**Affiliations:** ^1^Department of Psychology, Rikkyo UniversitySaitama, Japan; ^2^Department of Neurology, Showa University School of MedicineTokyo, Japan; ^3^Department of Design Science, Chiba UniversityChiba, Japan

**Keywords:** mutual interaction, joint action, perception-action coupling, prediction, synchronization, asymmetry, motion capture

## Abstract

Altering physical actions when responding to changing environmental demands is important but not always effectively performed. This ineffectiveness, which is an error of social behavior generated by mutual interactions, is not well understood. This study investigated mechanisms of a hesitant behavior that occurs in people walking toward each other, causing people to move in the same direction when attempting to avoid a collision. Using a motion capture device affixed to 17 pairs, we first confirmed the hesitant behavior by a difference between the experimental task, which involved an indeterminate situation to assess the actions of another individual, and the control task, which involved a predetermined avoiding direction, in a real-time situation involving two people. We next investigated the effect of three external factors: long distance until an event, synchronized walking cycle, and different foot relations in dyads on the hesitant behavior. A dramatic increase in freezing and near-collision behavior occurred in dyads for which the avoiding direction was not predetermined. The behavior related with the combination of long distance until an event, synchronized walking cycle, and different foot relations in dyads. We found that the hesitant behavior is influenced by an interpersonal relationship under enough distance to predict other movement. The hesitant behavior has possibly emerged as an undesired by-product of joint action. These results contribute to our understanding of the mechanisms of adaptive control of perception-action coupling in mutual interaction.

## Introduction

Appropriate control of the coupling of perceptions to actions leads to superior goal-directed motor behavior, and reflects an important psychosocial adaptation of conscious or unconscious control abilities (Aarts et al., 2008; Filevich et al., 2012; Land, 2012). Joint action can be defined as a social interaction whereby two people coordinate their actions with a co-representation of the action and its goal in mind (Sebanz et al., 2006). The joint action also requires coordination of one's actions with those of others in space and time and across different sensory modalities (Sebanz and Knoblich, 2009). Previous findings suggest that visual, auditory, and tactile targets are represented in a common visual reference frame that facilitates communication and integration of different sensory inputs and enables the translation into movement plans (Pouget et al., 2002; Harrar and Harris, 2010). More recently, studies have identified a behavioral dynamic of complementary collision-avoidance, using a task that involves moving targets without them colliding into each other on a computer screen (Richardson et al., 2015). Richardson et al. (2007) also showed that an interpersonal coordination in rocking chair movements is constrained by the self-organizing dynamics of coupled oscillator systems.

However, such systems are not always conducive to positive outcomes. In an active social situation, people moving past each other on the street can unexpectedly move in the same direction in an attempt to avoid colliding, resulting in freezing or a near miss/collision (see Supplementary Video 1). We termed this phenomenon hesitant avoidance while walking (HAW). Although this behavior is a common experience, to our knowledge, its cause, which we hypothesize to be an error of social behavior generated by mutual interaction, has not been investigated.

One of the processes that could be involved in HAW is a predictive process. Predictive processes allow humans to understand others' intentions, and to anticipate what they will do next (Becchio et al., 2012). It is also thought that perception-action coupling and predictive processes functionally interact in humans on multiple levels (Gangopadhyay and Schilbach, 2012). Observed actions are processed as visual events that can be perceptually described, and as motor events represented in both time and space as a sequence of motor commands (Noy et al., 2011; Pizzolato et al., 2012; Nummenmaa et al., 2014). The processes can be engaged simultaneously, and information may be exchanged between them during perception-action and prediction (Keysers and Gazolla, 2007; Kilner et al., 2007). We first hypothesized that the prediction aspect is a main factor in HAW.

We then studied three environmental (external) factors potentially influencing HAW. The first was the distance until the event, which would increase the propensity to prediction by increasing the available time to predict (in correlation with increased distance) prior to the event. The second factor was a cycle synchronization function, which is an automatic arrangement of sensory motor synchronization among dyads (Richardson et al., 2007; Oullier et al., 2008). Evidence indicates that automatic synchronization in coupled dyads occurs in human behavior such as rocking (Richardson et al., 2007) and finger movements (Oullier et al., 2008). In an unexpected situation, the cycle synchronization of walking might influence the unintentionally implicit function. The third factor is human body/brain asymmetry (Nicholls and Roberts, 2002; Stephan et al., 2007). The asymmetric function, which reflects left-right hemispheric specialization, works not only on visual and auditory fields (Hellige, 1996; Lazard et al., 2012), but also on action dynamics (van den Berg et al., 2011). The positional relation of the participants' feet in a dyad, if the ipsilateral feet are the same at the avoiding moment, can increase the difficulty in choosing different avoidance directions due to the inherent asymmetries of the two persons. We hypothesized that these three external factors accelerate HAW, because the factors could unintentionally affect the instant prediction and prevent each person in the dyad from moving in a different direction.

To experimentally confirm and quantify the degree of HAW, we first calculated a difference between the experimental task which is an indeterminate situation involving the assessment of the actions of another individual and the control task which involves a predetermined avoiding direction. We defined the difference as the degree of HAW. The difference was measured in terms of a delay time (DT) for the time duration, and in terms of incidence of moving in a mistaken direction (MMD), which was defined as movements in a direction opposite to the ultimate course. The former and the latter reflect freezing and a near miss/collision. We next tested, for DT and MMD, whether three main effects of external factors (two free-zone distances, two walking-cycle synchronization types, and two starting feet relationships) and/or the 8 combinations among the three factors in interpersonal relationships increase HAW. The optimal prediction based on a top-down process is to move in the direction opposite to the other person (i.e., without HAW). In the absence of an available cue (the experimental condition), the predictive process should be strongly influenced by the interpersonal relationship, which is a bottom-up process. However, the prediction should not be influenced by the interpersonal relationship in the presence of an available cue (the control condition). We hypothesized that the automatic perception-action process, as a bottom-up process, facilitates confusion in the decision when neither person knows the other's action. Furthermore, the external factors mentioned above should also increase the confusion, and thus influence the probability of HAW occurring.

## Materials and methods

### Participants

A total of 36 university students between 20 and 23 years of age (20 females, mean age = 21.2 years, SD = 0.23) participated in the study. No participants had histories of drug or alcohol abuse or histories of neurological or psychiatric disorders. All participants had normal vision (corrected/uncorrected visual acuity, ≥0.7 on the Landolt ring chart), and all were right-foot dominant. The participants were randomly placed in same-gender pairs (female pairs: 10, male pairs: 7, chi-squared test: *X*^2^ = 0.529, *p* = 0.467) and were paired with strangers to avoid any potential gender or familiarity effects. Written informed consent was obtained from all participants prior to the study. The Research Ethics Committee of Rikkyo University reviewed and approved all experimental procedures, which complied with the Declaration of Helsinki guidelines.

### Procedures

**Figure 1:**
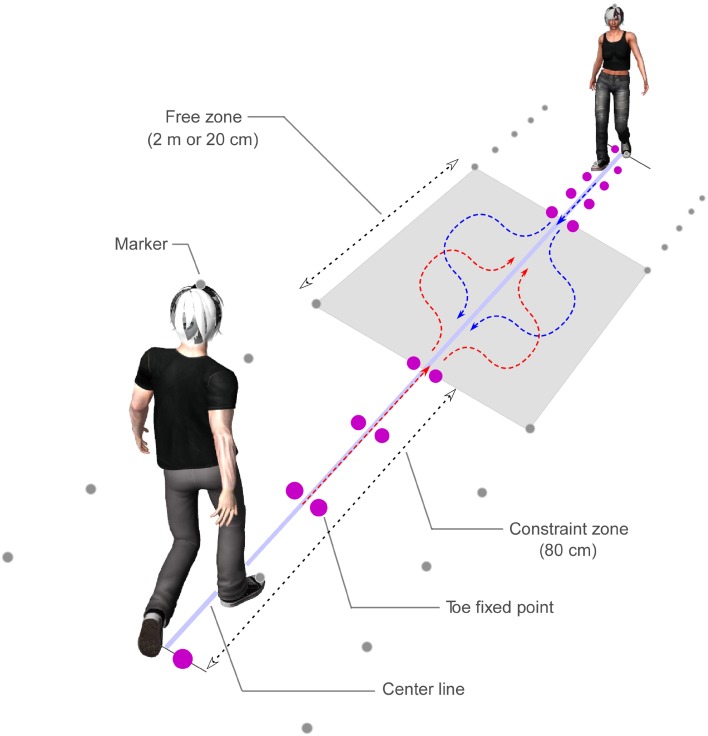
**Experimental schema of the walking task**. Participants wore wireless headphones, and capture markers on the tops of their heads and on their feet toes. Participants heard a timing sound that was presented at 500 ms intervals. The constraint zones were each 80 cm in length, and the free zone was 200 cm in the long condition, and 20 cm in the short condition. There were five fixed toeing points in the constraint zone on the right and left. In the synchronization condition, the timing sounds were presented at the same time to two persons, whereas in the asynchronization condition, mismatched sounds with 200 ms difference between the two participants were presented. In the starting foot side condition, the two participants began on the same or on opposite feet.

Participants wore wireless headphones and capture markers on the tops of their heads and on their feet and toes (Figure 1). An optical motion capture device (ProReflex, Qualisys, Sweden) recorded the markers at 60 Hz with spatial resolution of 1 mm. The participants heard a timing sound with a frequency of 1000 Hz that was presented at 500 ms intervals. A countdown voice that said, “three, two, one, zero” was broadcast to initiate the trial. The first step on the ground occurred with the “zero” of the countdown. Individuals were allowed to practice the start-timing of walking prior to testing. The constraint zones were each 80 cm in length, and the free zone (i.e., the distance until the event) was 200 (long) or 20 (short) cm in length. In the constraint zone, five fixed toeing points were located to each of the right and left, including the starting points. In other words, the participants reached the free zone in four steps. The ground was marked with a center line and capture markers.

Participants were required to (1) walk along the fixed toeing points in rhythm with the timing sounds, starting on the indicated foot, while looking at the other participant's face in the constraint zone; (2) avoid colliding with the other participant when they entered the free zone; (3) move to the center line as quickly as possible after passing the other participant; and (4) walk through the other participant's constraint zone independently of the fixed toeing points. The timing sounds were presented only in the constraint zone.

In the synchronization condition, the timing sounds were presented at the same time to each participant. In contrast, the timing sounds were presented to each participant with a gap of 200 ms in the asynchronization condition. In the starting foot side condition, the two participants began on the same or on opposite feet. That is, in the different condition, one participant began on the right foot while the other began on the left. The experimenter visually indicated the starting foot to each participant separately, and the participants did not know the starting foot of the other participant. The starting feet were pseudo-randomly determined for each pair. In the experimental task, combinations of eight conditions that included two walking-cycle synchronization types (SYNC; synchronization or asynchronization), two free-zone distances (DIST; long: 200 cm; short: 20 cm), and two starting feet relationships (FOOT; different or same feet) were created. In the control task, the experimenter indicated the avoidance direction to each participant before each trial, while the same eight conditions were presented as for the experimental task. All trials were counterbalanced within each pair.

### Data analysis

**Figure 2:**
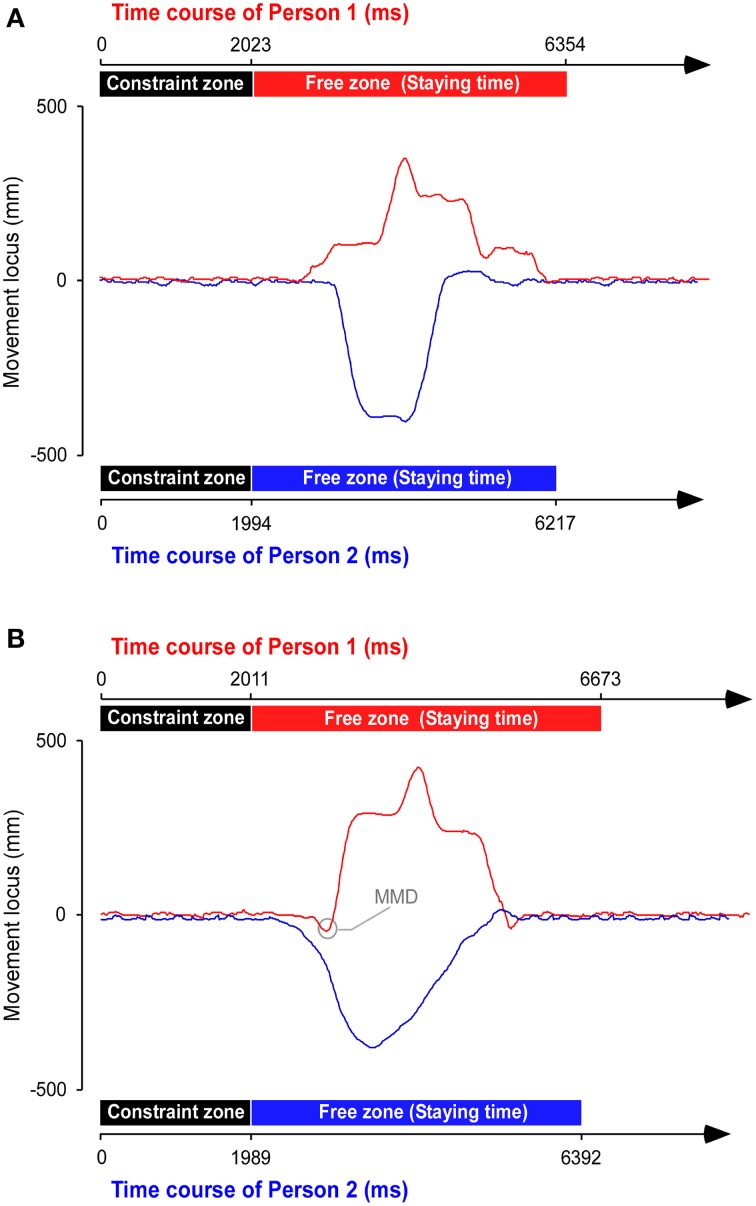
**Trajectory of a representative pair**. The example shows a trial in which the conditions are synchronization, long distance, and different starting feet of a pair (Person 1 and 2) in **(A)** control and **(B)** experimental tasks. The movement locus was simultaneously obtained from both persons. The time axis of Person 2 was inverted, and the start position was adjusted for Person 1. Note the high rate of moving in a mistaken direction (MMD), when a participant moved to one direction initially but ultimately moved to the other direction, in the experimental task.

The experimenter recorded the three-dimensional coordinate data obtained from all of the capture markers. In each trial, the staying time in the free zone was measured separately for the head markers of each participant, and these times were averaged. Using this staying time, DT was computed by subtracting the control staying time from that of the experimental task. The number of occurrences of MMD was also counted (Figure 2). A maximum horizontal oscillation during walking in the constraint zone was set as a standard in each person. The horizontal oscillations on one side with reference to the center line in constraint zone ranged from 8 to 25 mm (average: 15.1 mm, SD: 0.94) across all participants. If the horizontal oscillation from the center line in free zone exceeded the standard when a participant moved to one direction initially, but ultimately moved to the other direction to avoid a collision, we counted it as MMD. MMD counts were calculated as the sum between both participants in each trial.

For the feet toe markers within the constraint zone, the initial and ending timing errors were all below 60 ms (average: 36 ms, SD: 2.9) in the synchronization condition. The time lag between the two participants was appropriately 200 ms (average: 203 ms, SD: 31) in the asynchronization condition.

### Statistical analysis

Paired *t*-tests were used to examine the differences in mean times between tasks in the 200- and 20-cm free zones. Unpaired *t*-tests were used to examine the sex difference in DTs. A Three-Way repeated-measures ANOVA was used to examine the main effects (2 SYNC × 2 DIST × 2 FOOT) and the interactions, and a multiple comparisons with Bonferroni corrections were used for DTs and MMD counts. All tests were two-tailed, and all results are presented as the means, standard errors of the mean, and effect size (η^2^). The level of statistical significance was defined as 0.05. SPSS version 20.0 (IBM, Inc.) was used for the statistical analyses.

## Results

### Indeterminate situation for the other's avoidance action causes HAW

**Figure 3:**
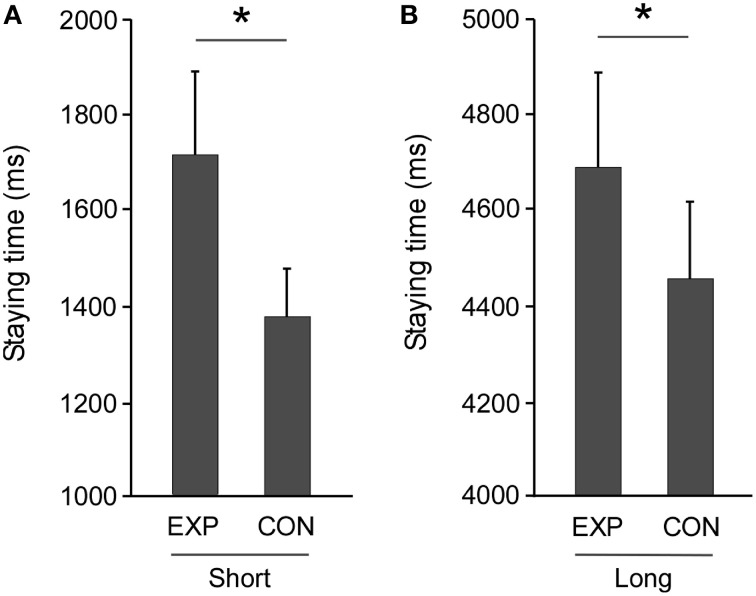
**Extension of staying time in an indeterminate situation for another's action**. A *t*-test showed that mean staying time in the experimental task (EXP) was significantly longer than that in the control task (CON) under **(A)** Short (20 cm in length) and **(B)** Long (200 cm in length) distance conditions (^*^p < 0.0001, respectively). Error bars indicate the standard errors of the mean.

The mean staying time, or the time spent in the free zone, in the 20-cm in length (short DIST) condition in the experimental task was significantly longer than that in the control task [*t*_(16)_ = 4.895, *p* < 0.0001, Figure 3A], and similar results were obtained for the experimental task with the 200-cm in length (long DIST) free zone [*t*_(16)_ = 4.454, *p* < 0.0001, Figure 3B]. Prior to analysis of external effects, we analyzed the difference between experimental and control tasks as DT. The DTs showed no significant sex difference in the long DIST [*t*_(15)_ = 1.488, *p* = 0.157] and the short DIST [*t*_(15)_ = 1.786, *p* = 0.094]. Furthermore, there were 57 MMDs in the experimental task out of 272 potentials (for 34 persons), whereas there were none in the control task out of a potential 272. No physical contacts between the participants occurred in any trial. Again, the MMDs showed no significant sex difference in the long DIST [*t*_(15)_ = 1.265, *p* = 0.225] and the short DIST [*t*_(15)_ = 1.213, *p* = 0.244].

### The confluence of factors extends freezing time

**Figure 4:**
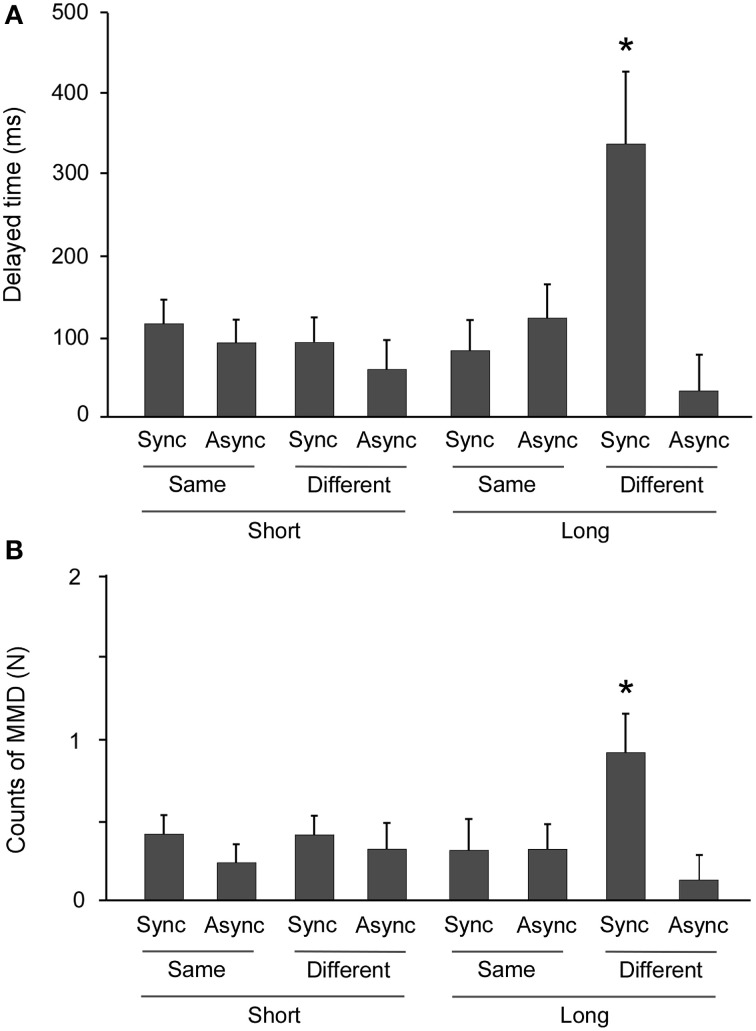
**Prolongation of freezing time and incidence of near collision**. An ANOVA revealed highly significant Three-Way interaction effects in both delay time (DT) and incidents of moving in a mistaken direction (MMDs). **(A)** Multiple comparisons tests revealed that DTs were markedly longer in only the combination of synchronization, long distance, and different starting foot conditions (^*^: all *p* < 0.05). **(B)** Multiple comparisons tests revealed that the MMD count was higher only when the conditions of synchronization, long distance, and different starting feet were combined, compared to all other conditions (^*^: all *p* < 0.05). Error bars indicate the standard errors of the mean.

A Three-Way analysis of variance (ANOVA) of DTs revealed a significant main effect of SYNC [*F*_(1, 16)_ = 12.441, *p* = 0.003, η^2^ = 0.437], and no significance in the main effects of DIST [*F*_(1, 16)_ = 2.393, *p* = 0.141, η^2^ = 0.130] and FOOT [*F*_(1, 16)_ = 1.898, *p* = 0.187, η^2^ = 0.106]. The interactions of SYNC × DIST [*F*_(1, 16)_ = 2.051, *p* = 0.176, η^2^ = 0.111], SYNC × FOOT [*F*_(1, 16)_ = 4.085, *p* = 0.060, η^2^ = 0.203], and DIST × FOOT [*F*_(1, 16)_ = 1.457, *p* = 0.245, η^2^ = 0.083] were likewise not significant. However, the SYNC × DIST × FOOT interaction was significant [*F*_(1, 16)_ = 8.821, *p* = 0.009, η^2^ = 0.355]. A multiple comparisons tests revealed that only the combined conditions of synchronization, long distance, and different feet produced longer DTs compared to other conditions (all *p* < 0.05) (Figure 4A).

### The confluence of factors increases the probability of a near collision

ANOVA of MMD counts in the experimental task (equals the difference between control and experimental tasks) revealed that the main effect of SYNC [*F*_(1, 16)_ = 8.544, *p* = 0.010, η^2^ = 0.348] was significant, and that the main effects of DIST [*F*_(1, 16)_ = 1.210, *p* = 0.288, η^2^ = 0.070] and FOOT [*F*_(1, 16)_ = 0.541, *p* = 0.473, η^2^ = 0.033] were not significant. The SYNC × DIST [*F*_(1, 16)_ = 0.704, *p* = 0.414, η^2^ = 0.042], SYNC × FOOT [*F*_(1, 16)_ = 1.766, *p* = 0.203, η^2^ = 0.099], and DIST × FOOT [*F*_(1, 16)_ = 1.531, *p* = 0.234, η^2^ = 0.087] interactions were not significant, but the SYNC × DIST × FOOT interaction was significant [*F*_(1, 16)_ = 4.857, *p* = 0.043, η^2^ = 0.233]. The multiple comparisons tests again revealed that only the combination of synchronization, long distance, and different foot conditions produced higher MMD counts compared to the other conditions (all *p* < 0.05) (Figure 4B).

## Discussion

The prediction effect promoted a clearly extended DT and greater incidence of MMDs. Furthermore, DTs and MMD counts peaked with the combination of long pre-event distance, synchronized cycle, and different foot conditions, suggesting that the confluence of the three studied external factors affected the increases in freezing and near misses. While the predictive process always depended on automatic perception-action processing in the experimental conditions, confusion in the predictive process should be augmented by the external factors. Consequently, the aforementioned confusion then facilitates the likelihood of HAW occurring. Moreover, in the case of MMD occurrence, after one person had previously moved toward a direction, the other sympathetically moved toward the same direction (see Figure 2B). There were obvious duration differences between the two persons in the timing of onset of the horizontal movement in the 57 MMD data (average: 509 ms, SD 98 ms). These results suggest that HAW was caused by entrainment (if one moves, the other follows). Regarding the behavioral dynamics of complementary collision-avoidance (Richardson et al., 2015), and of coupled oscillator systems (Richardson et al., 2007), the bottom-up systems seem to produce a positive interpersonal action, such as synchronization or entrainment. However, the interpersonal relationship in our real-time walking task, which is a bottom-up process, generated in the constraint zone affected the predictive process in the free zone in the absence of an available cue. The resulting increase in confusion should be increased the likelihood of HAW occurring as a negative interpersonal action.

Probabilistic predictions for the future outcomes of actions by one's self and others are computed using previously learned action-outcome mapping (Schultz and Dickinson, 2000; Mathys et al., 2011). The reward prediction errors that are generated in dopaminergic neurons are thought to encode the magnitude of the discrepancy between expected and experienced rewards (Schultz and Dickinson, 2000; Mathys et al., 2011; Friston, 2012). Error processing, response monitoring, and cognitive control are intrinsic to predictive processing (Hoffmann and Falkenstein, 2012), and the dorsal anterior cingulate cortex provides continuously updated predictions of the expected cognitive demand by optimizing future behavioral responses (Sheth et al., 2012). The dynamic changes in neural activity that occur during the preparation and imagination of one's own movements correspond at least in part to the neural processes recruited for prediction of action kinematics and action understanding during the observation of others in social interaction (Grèzes and Decety, 2001). In HAW, the avoidance hitting others should be the optimal behavior based on the reward process at the time. HAW was heightened in the long pre-event duration, demonstrating that the long distance must have created prediction error. In other words, the short distance should reduce the prediction error. We suggest that the confusion in the predictive process generated by the long distance facilitated HAW. On the other hand, participants mechanically selected the most appropriate action when they had no time to avoid the other person.

Walking cycle synchronization in dyad strongly affected HAW. Spontaneous synchrony is achieved unconsciously, without engaging higher cognitive processes or particular action goals. This automatic synchronization in coupled dyads has been observed in human behavior, such as rocking and finger movements (Richardson et al., 2007; Oullier et al., 2008), as well as in specific animal behaviors like button pressing (Nagasaka et al., 2013). The activation of apparently functionally specific mirror neurons in the premotor cortex during action execution, action observation, and the formation of action intentions (Gallese et al., 1996; Fogassi et al., 2005; Caggiano et al., 2009) has led to numerous suggestions of a role for these neurons in perception-action coupling. Given that the mirror system is recruited during action observation, unintentionally synchronized walking cycles could facilitate the failure of the brain's collision avoidance systems. For example, action plans and intentions of observed actions can modulate event-related potentials that are associated with the early visual processing of the observed actions (Bortoletto et al., 2011). Furthermore, data indicate that passively observing a task-irrelevant, rhythmical action biases the cycle time of a subsequently executed rhythmical action (Eaves et al., 2014). Additionally, synchronized/communicative interactions can even influence visual discrimination (Neri et al., 2006) and the detection of biological motion (Manera et al., 2011). It has also been shown that individual differences in the ability to make temporal predictions of forthcoming events are notable under conditions of interpersonal sensorimotor synchronization (Pecenka and Keller, 2011; Schmidt et al., 2011). Some researchers have claimed that people actively and mutually adapt to each other's behavior in order to synchronize their movements (Konvalinka et al., 2010). Together, these findings demonstrate an early effect of perception-action coupling on an unconscious processing that occurs at an early stage of processing and prior to awareness. Thus, cycle synchronization in an unexpected situation might influence an implicit function, and as a result, synchronized feet should affect a predictive process and then facilitate HAW.

The positional relation of the feet also plays a supplementary role in HAW. Humans and other higher primates share a left hemispheric specialization for action dynamics, and a parietofrontal network that is larger in the right hemisphere than in the left (Stephan et al., 2007). In fact, the degree of anatomical lateralization and asymmetry is correlated with performance on a visuospatial task and in language processing (Day and MacNeilage, 1996; Siman-Tov et al., 2007). Hemispheric specialization is also associated with unbalanced processing speeds (Thiebaut de Schotten et al., 2011). In humans, turning asymmetries are biased to the right side, which suggests a commonality with left hemispheric intentional control (Stephan et al., 2007). The leftward perceptual bias could be the result of left-to-right scanning biases and premotor activation of the right hemisphere (Nicholls and Roberts, 2002). A transcranial magnetic stimulation study revealed a hemispheric asymmetry in which ipsilateral motor responses were larger when elicited in the left primary motor cortex compared with those elicited in the right (van den Berg et al., 2011). At the avoiding moment, with HAW it should be more difficult to choose different avoidance directions, due to the asymmetries of the two persons, if the ipsilateral feet are the same. The asymmetry can potentially affect the cycle synchronization during walking, and subsequently should also affect a predictive process and then facilitate HAW.

The study has several limitations. The results demonstrate an error of social behavior generated by mutual interaction within an experimental environment, while this method excludes social contextual effects such as gaze, social norm, or gender. We instructed the participants to look at the other participant's face in the constraint zone because gazing at the face or foot should strongly affect HAW; however, the gaze recording were not performed. Social norms such as walking or driving “on the right-hand” could also be a factor for HAW, and it is important to consider cultural difference. Furthermore, although in the current study, gender differences of each pair did not affect the HAW, it is possible that inter-gender interactions act as a confounder. Further, research is required to elucidate whether our reported effects are due to these high-level strategies. Finally, we set up only 2 types of distances for the free zone, and if we set up 110 cm or more in detail distance conditions, it may lead to a function curve for more appropriate interpretation of HAW. Future research also requires more advanced settings for maximal efficacy of HAW.

Subjectively experienced error in HAW is the first aspect to receive significant scientific focus. Joint action plays a fundamental role in human life and it requires the coordination of one's actions with those of others (Sebanz et al., 2006; Sebanz and Knoblich, 2009). Indeed, coordinated and cooperative actions are particularly relevant to social interaction and scenarios (Müller et al., 2011). It is possible that HAW has emerged as an undesired by-product of joint action consisting of complementary and coupled oscillator systems. This error of mutual social behavior should contribute to our understanding of the mechanisms of adaptive control of perception-action coupling in changing social environments. In addition, it has the potential for identifying ideas for preventing serious accidents related to these mutual interactions.

## Author contributions

MH designed the study and performed the experiment. MH and SK developed analytical tools and analyzed the data. All authors discussed the results. MH wrote the initial manuscript. All authors edited the manuscript.

### Conflict of interest statement

The authors declare that the research was conducted in the absence of any commercial or financial relationships that could be construed as a potential conflict of interest.

## Supplementary material

Supplementary Video 1 **Example of hesitant avoidance while walking (HAW)**. People moving in the same direction in an attempt to avoid colliding with each other on a street, and the consequent occurrence of freezing behavior or a near collision was observed. The latter half of the video is zoomed in. The video is a view from above a pedestrian overpass in Tokyo city. In the 4 h from 4 to 8 p.m., 10 samples were observed in the fixed frame.
